# QTc interval-dependent body posture in pediatrics

**DOI:** 10.1186/s12887-020-1959-8

**Published:** 2020-03-06

**Authors:** Björn Reynisson, Gustaf Tanghöj, Estelle Naumburg

**Affiliations:** Institution of Clinical Science, Pediatrics, Umeå University, Umeå, Sweden

**Keywords:** Child, Electrocardiography, Long QT-syndrome, Pediatrics, QTc, Standing test, Syncope

## Abstract

**Background:**

Syncope is a common and often benign disorder presenting at the pediatric emergency department. Long-QT syndrome may be presented with syncope, ventricular arrhythmias or sudden death and is vital to exclude as an underlying cause in children presented with syncope. Few studies have assessed QTc in relation to body posture in children. In this study, we assessed the QTc interval while laying down and during active standing in children with known long-QT syndrome compared to healthy controls.

**Methods:**

Children aged 1–18 years with long-QT syndrome (*N* = 17) matched to two healthy controls (*N* = 34) were included in this case-control study. The ECG standing was performed immediately after the ECG in the supine position. The QTc interval and QTc-difference by changing the body position were calculated.

**Results:**

All children with long-QT syndrome were treated with propranolol. QTc was prolonged among long-QT syndrome children while lying down and when standing up, compared to controls. A prolongation of QTc appeared when standing up for both cases and controls. There was no significant difference in QTc increase between the groups. A QTc over 440 ms was observed among four cases lying down and in eight cases while standing, but not in any of the controls. The standing test with a cut-off of 440 ms showed a sensitivity of 47% and a specificity of 100% for case-status in our study.

**Conclusion:**

QTc measured on ECG when rapidly rising up is prolonged in both healthy and LQTS children. More importantly, it prolongs more in children with LQTS and increases in pathological levels.

## Background

Syncope is a common condition in the pediatric population [1]. The underlying cause of syncope is often benign and often explained by orthostatic intolerance or vasovagal reactions [2, 3]. However, some life-threatening diseases may present as syncope during childhood [2, 4, 5].

Long QT syndrome (LQTS) is an uncommon genetic cardiac electrophysiological disorder in which altered ion channels within the myocytes cause prolonged repolarization [6]. This alteration may induce Torsades de Pointes (TdP) and lethal ventricular tachyarrythmias [7]. LQTS often presents as QT-prolongation and T-wave abnormalities on an electrocardiogram (ECG) [6, 8]. Congenital LQTS is an autosomal dominant hereditary disease caused by mutations in one or several known genes that regulate the function of ion channels of the myocytes [9]. The most common types found in genotyped cases are mutations associated with LQT1, LQT2 and LQT3 [6, 10]. Genetic testing is available and a routine procedure in known families and is used in the evaluation of malignant syncope [11–13]. According to national guidelines, prophylactic treatment with propranolol is recommended for all children with LQTS in Sweden [14].

The QT-interval in ECG is suggested to be adjusted to heart rhythm, QT-corrected time (QTc). In children 1–15 years of age, a QTc > 440 ms is considered to be the borderline upper limit of the QTc interval, while QTc > 460 ms is considered prolonged according to the national guidelines [14]. Over 25% of genetically evident LQTS have a normal ECG, and 10–15% of the general adult population has a borderline QTc [7, 15]. Thus, ECG combined with medical and hereditary history is included in the evaluation of children at risk [16, 17]. Genetic testing is not first in line in patients with syncope admitted to the pediatric department. Life-threatening cardiac causes must be ruled out by ECG, exercise testing (EST) and/or 24-h ECG [18]. EST and 24-h ECG are costly or not possible in younger children and/or are often associated with a long waiting list. There is a need for an easily available method when assessing malignant syncope in children [19].

Postural changes are known to increase heart rate, heart rate variability and syncope [20, 21]. A significant change in QT-interval and QTc interval when standing is seen in adults with known LQTS compared to controls [22, 23]. An increase in QTc-interval among healthy children when standing has been shown in a few studies, indicating the need for more research [24–26]. No studies have compared QTc alterations in children with LQTS compared to healthy controls.

We hypothesized that a standing test could be valuable in the evaluation of QTc in children. Furthermore, we hypothesized that QTc measured while standing is prolonged in children with LQTS compared to otherwise healthy children.

## Methods

### Materials

This pilot case-control study included children between 1 and 18 years of age with genetically diagnosed LQTS attending the Pediatric Cardiology department at Östersund Hospital, Sweden. Two healthy cardiac controls matched by age and gender were randomly selected from the pediatric department while attending the clinic for other reasons.

### Method

A standard 12-lead ECG was conducted in a rested supine body position and followed by an ECG immediately after standing up. The children rested in the supine position for as long as needed for a reliable ECG. The ECG were conducted in a supine position and immediately followed by active standing. Information on age, height, weight, other medical conditions, medication, blood pressure, history of syncope or family members with known LQTS was retrieved for cases as well as controls. Drugs were stratified into four groups: 1) beta-blockers, 2) drugs that can cause QT prolongation or induce TdP, 3) other medications and 4) occasional medications. The “List of drugs to be avoided by congenital LQTS patients” available at www.crediblemeds.org was used for classification.

### ECG measures

*The* QT interval was manually measured predominantly in lead II or using other leads when the quality of lead II was low. The QT-interval was measured in milliseconds (ms) from the beginning of the QRS-complex to the end of the T-wave using the tangent method to locate the end of the T-wave [27]. Bazett’s formula was used for QTc calculations [28]. QTc was measured for cases and controls in supine follow by a standing body position. Three medical doctors calculated QTc intervals individually, and two of these doctors were blinded with regard to case and control status as well as to body position. The mean QTc value was calculated between these separate measurements. A standing test using a cut-off value of 440 ms to define risk for LQTS was performed. Specificity and sensitivity were calculated for the standing test to identify LQTS cases among cases and controls.

### Statistical analysis

Interobserver variability of ECG measurements was calculated using Cornbach’s analysis. All data are presented as the mean (SD), median (range) or percentage (%) depending on the type and distribution of the data. Continuous data were primarily tested for normality using the Shapiro-Wilks test. Student’s t-test (paired two-sided) was used for parametrically distributed variables within groups, One-way ANOVA between the groups, Repeated measures ANOVA for QTc changes and Person’s X^2^ or Fisher’s exact test for categorical data according to fit, with *p* < 0.05 considered to be significant. All statistical analyses were performed using SPSS, version 25.

## Results

### Study-group demographics

**Fig. 1:**
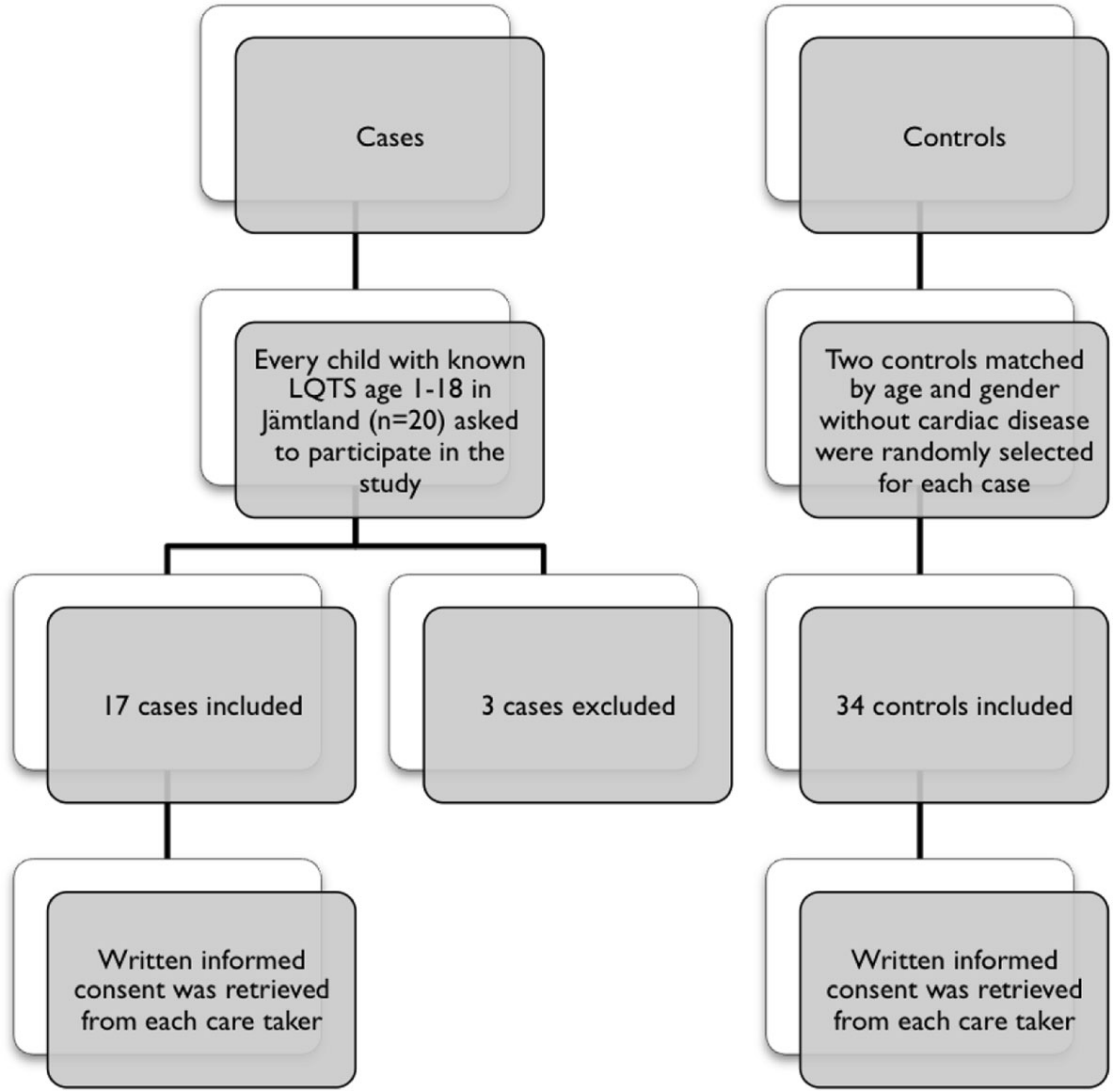
Study population

Overall, 20 children were identified with LQTS at the pediatric cardiac departments. Two children were excluded due to incapacity to follow instructions due to young age and one declined participation, leaving 17 children to be included in the study (Fig. 1). Two cases were randomly matched to each case, including a total of 51 children in the study (17 cases and 34 controls).

The mean age for cases was 9.0 years (±4.2 years), and for controls, 9,4 years (±4.2 years) and 76% of study participants were less than 12 years of age. There was no difference between cases and controls with regard to gender distribution, age, height, weight, history of syncope, other diseases or noncardiac medications (Table 1). Treatment with beta-blockers, a lower blood pressure and a heredity for LQTS was more common among cases compared to controls (Table 1).

**Table 1:** Population characteristics – demographics

Variables	Cases *N* = 17	Controls *N* = 34	*p*
Weight mean (kg)	35.0 (SD 22.3)	38.8 (SD 20.8)	0.552
Height mean (cm)	134.2 (SD 24.9)	136.6 (SD 24.4)	0.745
Female n (%)	9/17 (53%)	18/34 (53%)	1.00
Male n (%)	8/17 (47%)	16/34 (47%)	1.00
Age mean (years)	9.0 (SD 4.2)	9,4 (SD 4.2)	0.724
Beta-blocker therapy n (%)	17/17 (100%)	0/34 (0%)	< 0.001
Systolic BP mean (mm Hg)	100.7 (SD 8.3)	109.1 (SD 13.3)	0.021
Diastolic BP mean (mm Hg)	56.0 (SD 10.5)	69.8 (SD 12.2)	< 0.001
Medications on LQTS avoid list n (%)	4/17 (23.5%)	3/34 (8.8%)	0.203
Other medications n (%)	4/17 (23.5%)	16/34 (47.0%)	0.135
Occasional medications n (%)	3/17 (17.6%)	12/34 (35.2%)	0.328
Heredity for LQTS n (%)	17/17 (100%)	0/34 (0%)	< 0.001
History of syncope n (%)	1/17 (5.8%)	2/34 (5.8%)	1.00
Asthma n (%)	6/17 (35%)	6/34 (17%)	0.181
Diabetes n (%)	0/17 (0%)	5/34 (14.7%)	0.156
Other diseases n (%)	3/17 (17.6%)	9/34 (26.4%)	0.728

*BP* Blood pressure, *LQTS* Long-QT syndrome

### QTc interval and body position

Interobserver analysis of QTc measurements showed an interclass correlation level of 0.855 (*p* < 0.001) for QTc in the supine position and 0.884 (*p* < 0.001) for QTc in the standing position.

**Fig. 2:**
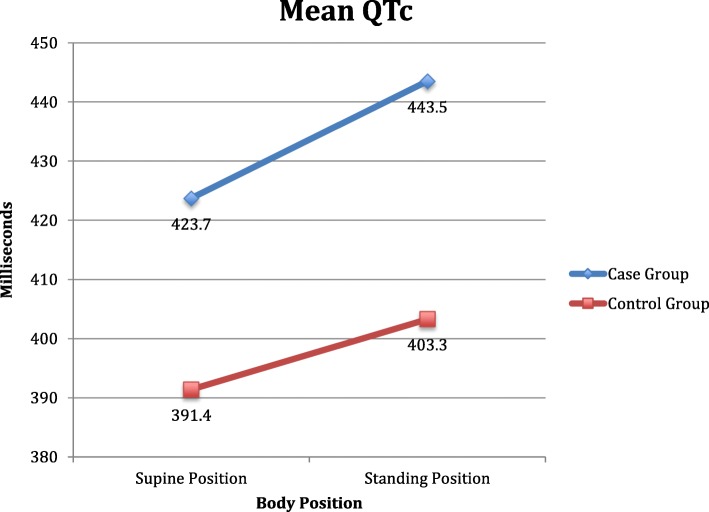
Mean QTc change from supine body position to standing among cases and controls

**Fig. 3:**
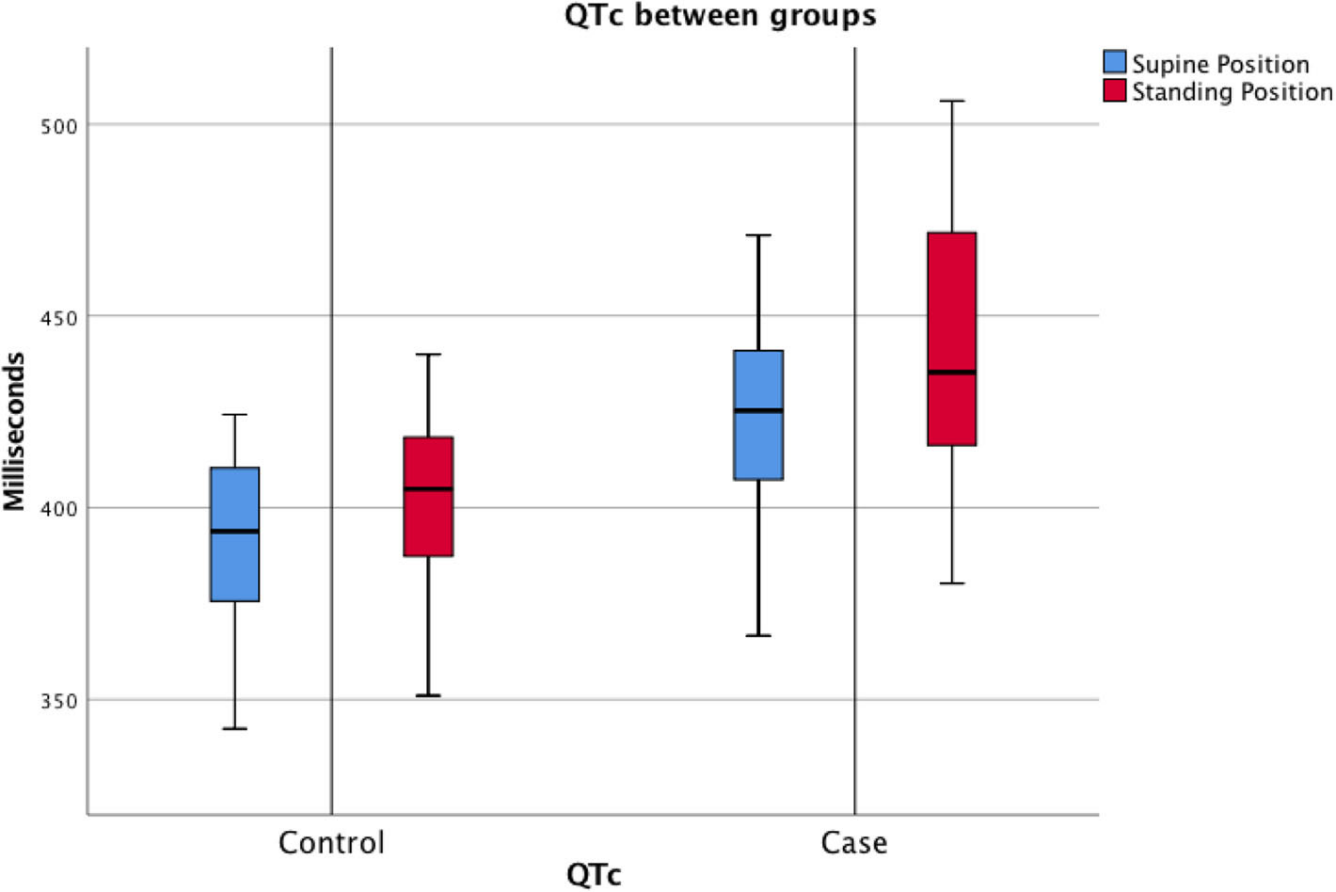
QTc median, range and percentiles of QTc for cases and controls

The mean QTc interval in the supine position was longer for cases (423.7 ms) compared to controls (391.4 ms) (*p* < 0.001) (Table 2, Figs. 2 and 3). The QTc interval was longer for cases (443.5 ms) compared to controls (403.3 ms) (*p* < 0.001) while standing (Table 2, Figs. 2 and 3), with a significant increase (Table 3). There was no difference in QTc-interval change between cases and controls in the supine position compared with standing (*p* = 0.308) (Table 2). A QTc interval of over 440 ms was calculated in four (24%) at the supine position and in eight (47%) while standing, but not in any of the controls (*p* = 0.003) (Table 2).

**Table 2:** QTc compared between groups

QTc between groups	Case group (*N* = 17)	Control group (*N* = 34)	Difference	*p*
QTc in supine position mean (ms)	423.7 (SD 27.4)	391.4 (SD 22.4)	32.3	< 0.001
QTc in standing position mean (ms)	443.5 (SD 39.5)	403.3 (SD 21.3)	40.2	< 0.001
QTc change mean (ms)	19.7 (SD 24.5)	11.9 (SD 26.7)	7.8	0.308
QTc > 440 m in supine position n (%)	4/17 (23.6%)	0/34 (0%)	4	0.003
QTc > 440 m in standing position n (%)	8/17 (47.0%)	0/34 (0%)	8	< 0.001

*Ms* milliseconds, *SD* Standard deviation

**Table 3:** QTc compared within groups

QTc change within groups	Supine position	Standing position	Difference	*p*
Case group mean (ms)	423.7(SD 27.4)	443.5 (SD 39.5)	19.7	**0.004**
Control group mean (ms)	391.4 (SD 22.4)	403.3 (SD 21.3)	11.9	**0.014**

*Ms* milliseconds, *SD* Standard deviation

A significant change in QTc mean from supine to standing position for case- and control-group.

### Standing test

The standing test was able to identify LQTS at risk by ECG and reached a specificity of 100% and a sensitivity of 47.0%. The ability to identify a patient with LQTS on ECG while lying down reached a specificity of 100% and a sensitivity of 24% in our study.

## Discussion

This pilot case-control study assessed the QTc interval in children with LQTS compared to healthy controls. An increased QTc-interval was present in children with LQTS in supine and standing body positions compared with controls. The standing test detected eight cases with prolonged QTc > 440 ms, which was not observed in four of the children while in a supine position.

### Prolongation of QTc-interval in children and testing

A prolongation of QTc-interval in healthy children when standing is in line with other studies [24, 26]. According to the national guidelines, a QTc interval over 440 ms may indicate a risk of LQTS [14]. A QTc > 440 ms was observed in a large proportion of healthy children in other studies, while none of the healthy controls in our study presented a QTc > 440 ms [24, 26]. The difference in the standing test method used in our study compared to other studies may explain the shorter QTc among controls in our study. The ECG standing was performed immediately after the ECG in the supine position, while others used a continuation of repeated ECG conducted at one-minute intervals [24, 26]. We chose to use this simpler method to better mimic a clinical setting at the emergency department where a simple standing test can be used in patients admitted to the ward for syncope. The QTc interval changes in children are mainly dependent on the increased heart rhythm while standing [26]. One can speculate that the lack QTc increase in other studies in repeated ECG may have captured the maximum tachycardia level in healthy controls and LQTS patients with normal autonomic function, and QTc measurements in our study did not adjust the maximum heart rhythm [26].

Our study indicates that the standing test with a sensitivity of 100% may rule out the risk of misclassifying a healthy child with LQTS. However, the specificity was only 47%, and a standing test will never be sufficient to confirm the diagnosis of LQTS. We believe that the standing test, together with other EST and 24-h ECG, may be used to evaluate which patients are likely to benefit from genetic screening. Furthermore, a standing test such as this is easy to perform and can be a useful tool in the selection of patients at risk.

### Prolongation of QTc-interval in children; gender and age

Increased mean resting heart beat and a longer QTc interval are observed among premenopausal females, indicating that age as well as gender and hormonal levels influence electrophysical processes and characteristics [29, 30]. The standing test in adults provides diagnostic information as the QT interval increases in response to heart rate acceleration by standing [22]. Age has been correlated with postural heart rate, as a higher heart rate does not indicate an increase in orthostatic intolerance [31]. The study group in this study was young, predominantly in a prepubertal age group; 76% were under the age of 12. Information on growth and development, including Tanner stage, is missing in our study, and the study group was too small for age-specific calculations. Further studies on standing tests during different levels of growth may be helpful in discriminating borderline LQTS patients from healthy pre- and pubertal children.

### Confounding factors, limitations and strength of the study

Cases had a lower systolic and diastolic blood pressure compared to controls (Table 1). This discrepancy is likely due to the use of beta-blockers among cases [32]. It is possible that beta-blockers may mask some of the QTc prolongation among children with LQTS. A majority of patients with LQTS are treated with beta-blockers, which is recommended by the national guidelines [14]. Thus, it would be unethical to test QTc in LQTS patients without medications.

The controls were randomly selected and attended the pediatric department for other medical conditions and matched by age, gender and absence of heart disease. Matching two controls for each case increased the sample size and power of the study. There is a risk for selection bias, as our controls were visiting the hospital for other medical conditions that may influence the risk of prolonged QTc by the condition itself or by medication. There was no difference between cases and controls regarding age, gender, weight or medication other than beta-blockers; thus, we believe the risk of selection bias influencing our results is limited.

## Conclusions

This pilot study shows significant differences in the QTc interval between children with LQTS and healthy controls in both the supine and standing positions, with a significantly prolonged interval in children with LQTS. With a standing test, we were able to detect a prolonged QTC interval in four cases, which was not present in these children while in a supine body position. These results suggest that there are significant differences between children with LQTS and healthy controls and that the use of a standing test could be valuable for the evaluation of syncope when suspecting LQTS.

## Data Availability

The data that support the findings of this study are available from the authors upon reasonable request.

## Abbreviations

ECG: Electrocardiogram
EST: Exercise testing
LQTS: Long QT syndrome
ms: Milliseconds
